# Comment on “Survey on the Knowledge and Practices in Anorexia of Aging Diagnosis and Management in Japan” by Takagi et al.—The Authors' Reply

**DOI:** 10.1002/jcsm.13770

**Published:** 2025-03-05

**Authors:** Sahoko Takagi, Shosuke Satake, Ken Sugimoto, Masafumi Kuzuya, Masahiro Akishita, Hidenori Arai, Ivan Aprahamian, Andrew J. Coats, Tatiana Klompenhouwer, Stefan D. Anker, Hidetaka Wakabayashi

**Affiliations:** ^1^ Department of Nutrition Management Hospital, National Center for Geriatrics and Gerontology Obu Japan; ^2^ Department of Geriatric Medicine, Hospital National Center for Geriatrics and Gerontology Obu Japan; ^3^ Department of Frailty Research Research Institute, Center for Gerontology and Social Science, National Center for Geriatrics and Gerontology Obu Japan; ^4^ Department of General and Geriatric Medicine Kawasaki Medical University Okayama Japan; ^5^ Meitetsu Hospital Nagoya Japan; ^6^ Tokyo Metropolitan Institute for Geriatrics and Gerontology Tokyo Japan; ^7^ National Center for Geriatrics and Gerontology Obu Japan; ^8^ Division of Geriatrics, Department of Internal Medicine Jundiaí Medical School Jundiaí Brazil; ^9^ Heart Research Institute Sydney New South Wales Australia; ^10^ Society on Sarcopenia, Cachexia and Wasting Disorders Lausanne Switzerland; ^11^ Department of Cardiology (CVK) of German Heart Center Charité Institute of Health Center for Regenerative Therapies (BCRT), German Centre for Cardiovascular Research (DZHK) partner site Berlin, Charité Universitätsmedizin Berlin Germany; ^12^ Institute of Heart Diseases, Wroclaw Medical University Wroclaw Poland; ^13^ Department of Rehabilitation Medicine Tokyo Women's Medical University Hospital Tokyo Japan

We thank Dr Cui for the constructive suggestions regarding our manuscript [1, 2].

The study was a secondary analysis of data collected from healthcare professionals in Japan as part of the global unified survey [3] conducted by the Society on Sarcopenia, Cachexia, and Wasting Disorders (SCWD). We focused on the availability of opportunities for continuing education and the differences in awareness and responses to anorexia of ageing (hereinafter referred to as AoA). To avoid losing the subtle nuances of the responses to the survey questions, we presented the results using descriptive statistics and chi‐square tests. However, as Dr Cui suggested, adjusting for potential confounding factors underlying the responses can provide deeper insights into the relationship between the availability of educational opportunities and attitudes towards geriatric care. Accordingly, we have further conducted an analysis based on a multivariable regression model, the results of which are reported below.

We used a logistic regression model to analyse the responses to the following two AoA survey questions that were directly related to treatment: ‘Do you use tools or resources to address decreased appetite in geriatric patients?’ (Q15) and ‘Are you confident in recommending nutritional interventions for geriatric patients with decreased appetite?’ (Q17). To facilitate the analysis, responses to Q15 were categorized as follows: 0 for *yes, all of the time* and *yes, most of the time* and 1 for all other responses. For Q17, 0 was assigned to *strongly agree* and *agree*, whereas 1 was assigned to all other responses. We conducted binary logistic regression analysis using ‘access to educational opportunities’ as the explanatory variable (0 = *yes*, 1 = *no*). Potential confounding factors included the occupation (categorized into six groups: physician, nurse, registered dietitian, pharmacist, rehabilitation therapist and other professions) and workplace (categorized into four groups: hospital, nursing home, home health care or clinic and others). The results showed that regardless of occupation or workplace, the group without educational opportunities had a significantly higher odds ratio (OR) for ‘not using tools or resources to care for geriatric patients with decreased appetite’ (OR: 3.86; 95% CI: 2.72–5.46) (Table 1) and ‘not being confident in recommending nutritional interventions for geriatric patients with decreased appetite’ (OR: 2.38; 95% CI: 1.74–3.26) (Table 2). These results indicate that even when accounting for potential confounding factors such as occupation and workplace, continuing education in nutrition influences the ability to provide AoA‐related care.

**TABLE 1:** Bivariate logistic regression analysis of responses to the question: ‘I use tools and resources such as evidence‐based guidelines to care for my geriatric patients with anorexia’ (*n* = 870).

Factor	Odds ratio (95% CI)	*p*
Educational opportunities (0 = *yes*, 1 = *no*)	**3.86 (2.72–5.46)**	**< 0.001**
Health profession		
Physician	1.00 (reference)	
Registered dietitian	**0.40 (0.26–0.62)**	**< 0.001**
Nurse	1.15 (0.52–2.53)	0.740
Pharmacist	1.10 (0.60–1.99)	0.760
Rehabilitation therapist	0.93 (0.56–1.56)	0.790
Other professions	0.79 (0.37–1.69)	0.540
Practice setting		
Hospital	1.00 (reference)	
Nursing home	1.10 (0.63–1.92)	0.730
Home health care or clinic	1.00 (0.63–1.56)	0.990
Others	0.85 (0.49–1.48)	0.570

*Note:* The dependent variable was dichotomized based on the questionnaire responses: 0 = *yes, all of the time* or *yes, most of the time*; 1 = *rarely*, *no, I prefer to use my own clinical judgement*, *no*, *I am not aware of tools and resources that can be used to care for my geriatric patients with anorexia*, *no, I do not use such tools and resources because I do not have access to them* or *not applicable for my professional role/responsibility*. When using physicians as a reference, registered dietitians with continuing education opportunities were more likely to report using guidelines and other tools to care for appetite loss (shown in bold).

Abbreviation: CI, confidence interval.

**TABLE 2:** Bivariate logistic regression analysis of responses to the question: ‘I am confident in providing nutrition recommendations for geriatric patients with anorexia’ (*n* = 866).

Factor	Odds ratio (95% CI)	*p*
Educational opportunities (0 = *yes*, 1 = *no*)	**2.38 (1.74–3.26)**	**< 0.001**
Health profession		
Physician	1.00 (reference)	
Registered dietitian	**0.38 (0.22–0.66)**	**0.001**
Nurse	1.58 (0.85–2.93)	0.150
Pharmacist	**1.74 (1.09–2.77)**	**0.020**
Rehabilitation therapist	1.06 (0.68–1.66)	0.800
Other professions	1.17 (0.59–2.33)	0.650
Practice setting		
Hospital	1.00 (reference)	
Nursing home	1.29 (0.80–2.08)	0.300
Home health care or clinic	0.92 (0.61–1.38)	0.690
Others	1.12 (0.68–1.86)	0.660

*Note:* The dependent variable was dichotomized based on the questionnaire responses: 0 = *strongly agree* or *agree*; 1 = *neither agree nor disagree*, *disagree*, *strongly disagree* or *not applicable for my professional role/responsibility*. With physicians as the reference group, registered dietitians with continuing education opportunities were more confident in suggesting nutritional recommendations for older people with decreased appetite. In contrast, the opposite relationship was true for pharmacists (shown in bold).

Abbreviation: CI, confidence interval.

In global ageing, there is concern that decreased appetite in older adults (i.e., AoA) may simply be considered ‘a consequence of aging’. This could lead to a vicious cycle of exacerbating sarcopenia and frailty, eventually resulting in a need for long‐term care [4]. This study highlights the importance of improving and expanding continuous nutrition education programs, irrespective of occupation or workplace, to ensure the early detection of these issues and promote independence among older adults.

## Conflicts of Interest

M.A. received research funding from Eisai, Kracie Pharma, Mitsubishi‐Tanabe Pharma and Tsumura and lecture fees from Bayer HealthCare, Daiichi Sankyo, Toa Eiyo and Towa Pharmaceutical. I.A. has received consultancy fees from Pfizer. A.C. has received honoraria and/or lecture fees from AstraZeneca, Boehringer Ingelheim, Menarini, Novartis, Servier, Vifor, Abbott, Actimed, Arena, Cardiac Dimensions, Corvia, CVRx, Enopace, ESN Cleer, Faraday, Impulse Dynamics, Respicardia and Viatris. S.D.A. has received grants and personal fees from Vifor and Abbott Vascular and personal fees for consultancies, trial committee work and/or lectures from Actimed, Amgen, AstraZeneca, Bayer, Boehringer Ingelheim, BioVentrix, Brahms, Cardiac Dimensions, Cardior, Cordio, CVRx, Cytokinetics, Edwards, Farraday Pharmaceuticals, GSK, HeartKinetics, Impulse Dynamics, Novartis, Occlutech, Pfizer, Repairon, Sensible Medical, Servier, Vectorious and V‐Wave. He has been named co‐inventor of two patent applications regarding MR‐proANP (DE 102007010834 and DE 102007022367), but he does not benefit personally from the related issued patents.
